# Draft Genome Sequence of *Agrococcus* *baldri* Strain Marseille-P2731

**DOI:** 10.1128/genomeA.00015-17

**Published:** 2017-03-09

**Authors:** Pamela Afouda, Gregory Dubourg, Noemie Labas, Didier Raoult, Pierre-Edouard Fournier

**Affiliations:** a Unité de Recherche sur les Maladies Infectieuses et Tropicales Emergentes, UM 63, CNRS 7278, IRD 198, INSERM 1095, Institut Hospitalo-Universitaire Méditerranée-Infection, Faculté de Médecine, Marseille, France; b Special Infectious Agents Unit, King Fahd Medical Research Center, King Abdulaziz University, Jeddah, Saudi Arabia

## Abstract

*Agrococcus baldri* strain Marseille-P2731 was isolated from a Siberian permafrost specimen dated around 10 million years. The 3,021,022-bp genome of strain Marseille-P2731, with a 71.82% G+C content, includes 2,844 protein-coding genes, 72 toxin/antitoxin modules, nine bacteriocin-encoding genes, and 1,266 genes associated with mobilome.

## GENOME ANNOUNCEMENT

The genus *Agrococcus* Groth et al. (1996) was created in 1996 following the isolation of two *Agrococcus jenensis* strains. The type strain DSM 9580 was isolated from soil (1). Currently, the genus *Agrococcus* includes 10 species, including *A. jenensis* Groth et al. 1996, *A. baldri* Zlamala et al. 2002, *A. carbonis* Dhanjal et al. 2011, *A. casei* Bora et al. 2007, *A. citreus* Wieser et al. 1999, *A. jejuensis* Lee 2008, *A. jenensis* Groth et al. 1996, *A. lahaulensis* Mayilraj et al. 2006, *A. terreus* Zhang et al. 2010, and *A. versicolor* Behrendt et al. 2008 (http://www.bacterio.net/agrococcus.html). The species *A. baldri* was created in 2002. The type strain DSM 14215 was isolated from an air sample from the “Virgilkapelle” in Vienna (2). It is an aerobic, Gram-positive, and non-endospore-forming bacterium. Its cells are irregular and spherical. The major fatty acids detected for this strain were saturated branched structures (e.g., anteiso-C15:0, anteiso- C17:0, iso-C15:0, and iso-C16:0) (2). Herein, we describe the draft genome sequence of *Agrococcus baldri* strain Marseille-P2731 (CSUR P2731 = DSM 103061), isolated from a Siberian permafrost specimen dated around 10 million years.

Genomic DNA of *A. baldri* strain Marseille-P2731 was sequenced using the MiSeq technology (Illumina, Inc., San Diego, CA, USA) with the mate-pair strategy. Genomic DNA was quantified using a Qubit assay with the high sensitivity kit (Life Technologies, Inc., Carlsbad, CA, USA) at 107.3 ng/µl. The 1,793,290 paired reads were trimmed and assembled using the SPAdes software (http://cab.spbu.ru/software/spades/).

The tRNAscan-SE tool (3) was used to find tRNA genes, whereas ribosomal RNAs were found using RNAmmer (4). Open reading frames (ORFs) were predicted using Prodigal (5), with default parameters. The predicted bacterial protein sequences were searched against the Clusters of Orthologous Groups (COG) using BLASTP.

The genome of *A. baldri* strain Marseille-P2731 is 3,021,022 bp long, with 71.82% G+C content. It is composed of three scaffolds (composed of three contigs). Of the 2,903 predicted genes, 2,844 were protein-coding genes and 59 were RNAs (four complete rRNA operons, one additional 5S rRNA, and 47 tRNAs). A total of 2,053 genes (72.19%) were assigned a putative function (by COG or by NR blast). In addition, 99 genes were identified as ORFans (3.48%). The remaining 581 genes were annotated as hypothetical proteins (20.43%). Strain Marseille-P2731 contains 1,266 genes associated with the mobilome, 485 and 72 genes coding for virulence and toxin/antitoxin production, respectively, and only four genes larger than 5,000 nucleotides. No gene coding for resistance to antibiotics was identified.

Strain Marseille-P2731 was deposited in the Collection de Souches de l’Unité des Rickettsies (CSUR) (WDCM 875) under number P2731 and in the DSMZ under number DSM 103061.

### Accession number(s).

The 16S rRNA gene and genome sequence of *Agrococcus baldri* were deposited in GenBank under accession numbers LT558844 and FPEG01000000, respectively.
